# A pan-cancer analysis of potassium channel tetramerization domain containing 12 in human cancer

**DOI:** 10.1038/s41598-023-41091-8

**Published:** 2023-08-25

**Authors:** Pan Liu, Zhilan Liu, Qiankun Luo, Qiang Fu, Xu Zhang, Pengfei Yu, Shuai Zhou, Yingying Wang, Jiali Zhang, Song Chen, Hongwei Zhang, Qinghai Zhu, Tao Qin

**Affiliations:** 1 Department of Hepato-Biliary-Pancreatic Surgery, Zhengzhou University People’s Hospital (Henan Provincial People’s Hospital), No. 7 Weiwu Road, Jinshui District, Zhengzhou, 450000 China; 2 Henan University of Chinese Medicine, Zhengzhou, 450000 China; 3 Translational Research Institute, Zhengzhou University People’s Hospital, Zhengzhou, 450003 China; 4 Academy of Medical Sciences, Zhengzhou University, Zhengzhou, 450003 China; 5 Zhumadian Central Hospital, No. 747, Zhonghua Road, Zhumadian, 463000 China

**Keywords:** Cancer, Biomarkers

## Abstract

Abnormal expression of the potassium channel tetramerization domain containing 12 (KCTD12) is closely related to the occurrence and development of various tumors, but a pan-cancer analysis of KCTD12 has not yet been conducted. We explored the association between KCTD12 and more than 30 human malignancies using The Cancer Genome Atlas (TCGA) and Gene Expression Omnibus (GEO) databases. First, the mRNA and protein levels of KCTD12 were examined and their correlations with tumor stage and survival were explored. Second, we analyzed the infiltration of CD8^+^ and CD4^+^ T cells and cancer-associated fibroblasts in tumors and explored the correlation between KCTD12 expression and tumor cell stemness, genomic heterogeneity, and diagnostic specificity. Finally, we explored the molecular mechanisms associated with KCTD12 using KEGG/GO analysis. The results showed that KCTD12 mRNA and protein expression levels decreased in most tumors was significantly associated with the prognosis of tumor patients, and the phosphorylation level of KCTD12 decreased in several tumors, such as S200 and T196, pancreatic adenocarcinoma (PAAD), lung adenocarcinoma (LUAD), and breast invasive cancer (BRCA). The expression of KCTD12 was positively correlated with the degree of cancer-associated fibroblasts infiltration in cervical squamous cell carcinoma and endocervical adenocarcinoma (CESC), head and neck squamous cell carcinoma (HNSC), PAAD, and stomach adenocarcinoma (STAD). The relationship between KCTD12 expression and CD8^+^ and CD4^+^ T cell infiltration was also clarified. KCTD12 showed high diagnostic sensitivity for various types of tumors and may be involved in tumor cell biology by affecting tumor cell stemness, tumor burden, and other characteristics. Finally, we analyzed the molecular functions of KCTD12 and possible KEGG/GO signaling pathways. In this study, we developed a biological marker for diagnosis, prognosis, and immune infiltration of the pan-cancers.

## Introduction

γ-Aminobutyric acid (GABA) is the major inhibitory neurotransmitter in the mammalian central nervous system, acting on γ-aminobutyric acid type A receptors (GABA_A_R) and γ-aminobutyric acid type B receptors (GABA_B_R). GABA_B_R is not only important for cognition, nociception, and neuroprotection, but also plays a key role in neuropsychiatric disorders^1^. KCTD12 is an auxiliary subunit of GABA_B_R that induces rapid receptor desensitization by uncoupling G_βγ_ from effector channels, particularly G protein-coupled internal rectifier potassium channels^2^. KCTD12 increases the stability of receptors at the cell surface and interrelates between them to regulate G protein-gated inwardly rectifying K^+^ channels that affect cellular excitability^3^.

The development of channelopathies in cancer highlights the possible role of potassium channels in cell proliferation^4^. The KCTD12 mutation is relatively common in families, but most existing studies have focused on psychiatric diseases. For example, mutations in the human KCTD12 promoter region contribute to bipolar I disorder, and high levels of KCTD12 protein are associated with depression and schizophrenia^5^. In recent years, several studies reported the role of KCTD12 in malignant tumors. KCTD12 expression is positively correlated with the prognosis of patients with melanoma. Its down-regulation promotes melanoma cell proliferation and metastasis in vitro and in vivo^6^ and was associated with poor prognosis in BRCA and colon adenocarcinoma (COAD)^7^. Moreover, KCTD12 regulates tumor cell stemness characteristics through the ERK pathway^8^. However, to date, a pan-cancer analysis of KCTD12 based on clinical big data has not been reported.

In the present study, we used TCGA and GEO databases to conduct pan-cancer analysis of KCTD12 for the first time, which mainly included KCTD12 mRNA and protein expression in various tumors, as well as the association of genetic mutations with prognostic status, gene mutation, protein phosphorylation level, and immune cell infiltration to explore relevant cytological pathways.

## Results

### KCTD12 expression analysis data

**Figure 1:**
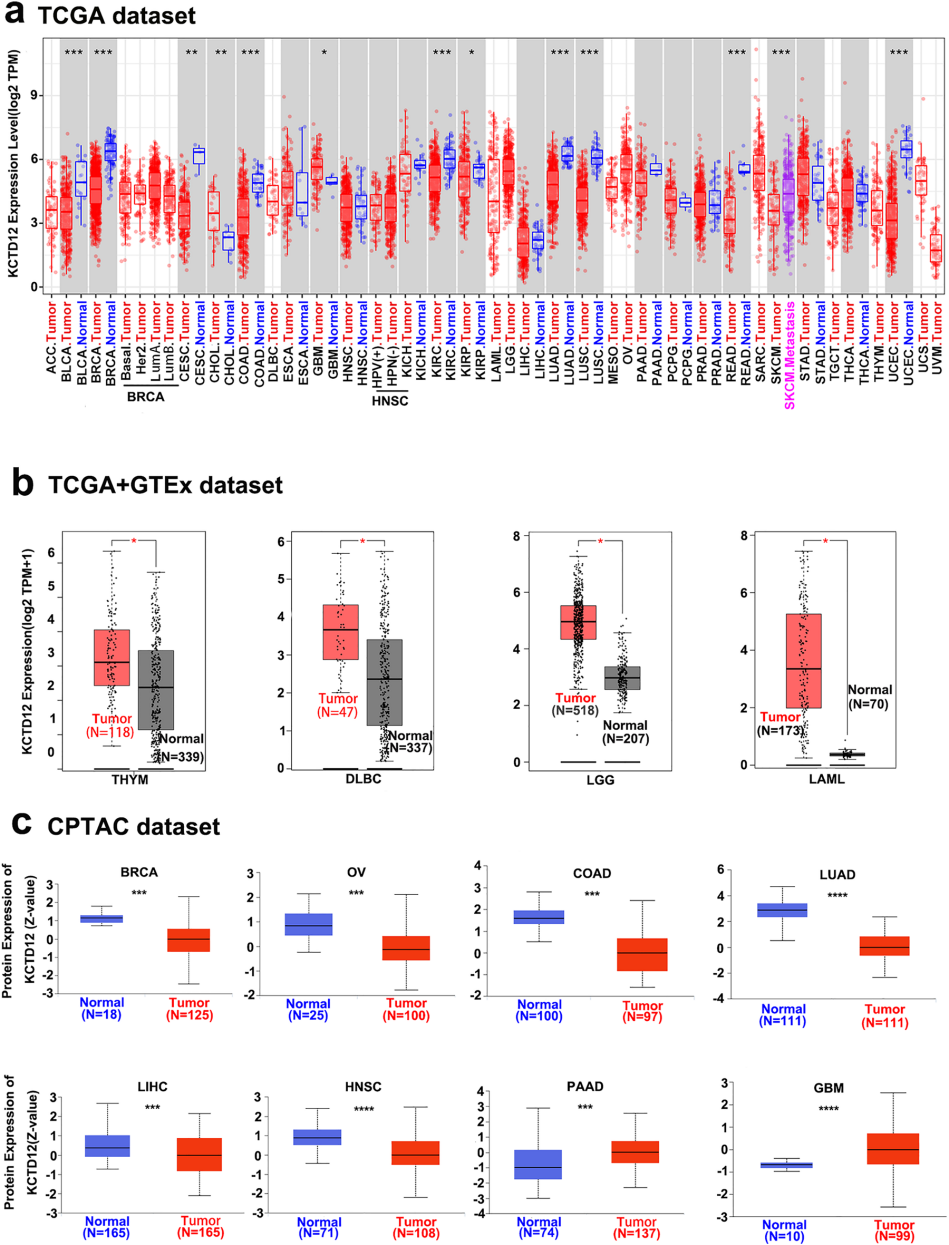
Pan-cancer KCTD12 expression levels. (**a**,**b**) Expression levels of KCTD12 mRNA in multiple tumors in TCGA and GTEx databases. (**c**) The expression levels of KCTD12 protein in various tumors in the CPTAC database.

The TIMER 2.0 portal combined with TCGA database showed differences in KCTD12 mRNA expression in various tumors. KCTD12 mRNA expression levels in bladder urothelial carcinoma (BLCA), BRCA, CESC, COAD, kidney renal clear cell carcinoma (KIRC), LUAD, lung squamous cell carcinoma (LUSC), rectal adenocarcinoma (READ), and uterine corpus endometrial carcinoma (UCEC) were significantly lower than those in normal tissues (*P* < 0.01). KCTD12 mRNA expression levels in cholangiocarcinoma (CHOL), glioblastoma multiforme (GBM), kidney renal papillary cell carcinoma (KIRP), lymphoid neoplasm diffuse large B cell lymphoma (DLBC), brain lower-grade glioma (LGG), and acute myeloid leukemia (LAML) were higher than those in normal tissues (*P* < 0.05) (Fig. 1a,b).

We explored the protein expression levels of KCTD12 in different tumors using the CPTAC database. The expression levels of KCTD12 protein in BRCA, ovarian serous cystadenocarcinoma (OV), COAD, LUAD, liver hepatocellular carcinoma (LIHC), UCEC, and HNSC tumor tissues were lower than those in the corresponding normal tissues (*P* < 0.001) (Fig. 1c) but were elevated in PAAD, GBM (*P* < 0.001) (Fig. 1c), and KIRC (*P* < 0.001) (Fig. S1).

### KCTD12 is associated with tumor stage and prognosis

**Figure 2:**
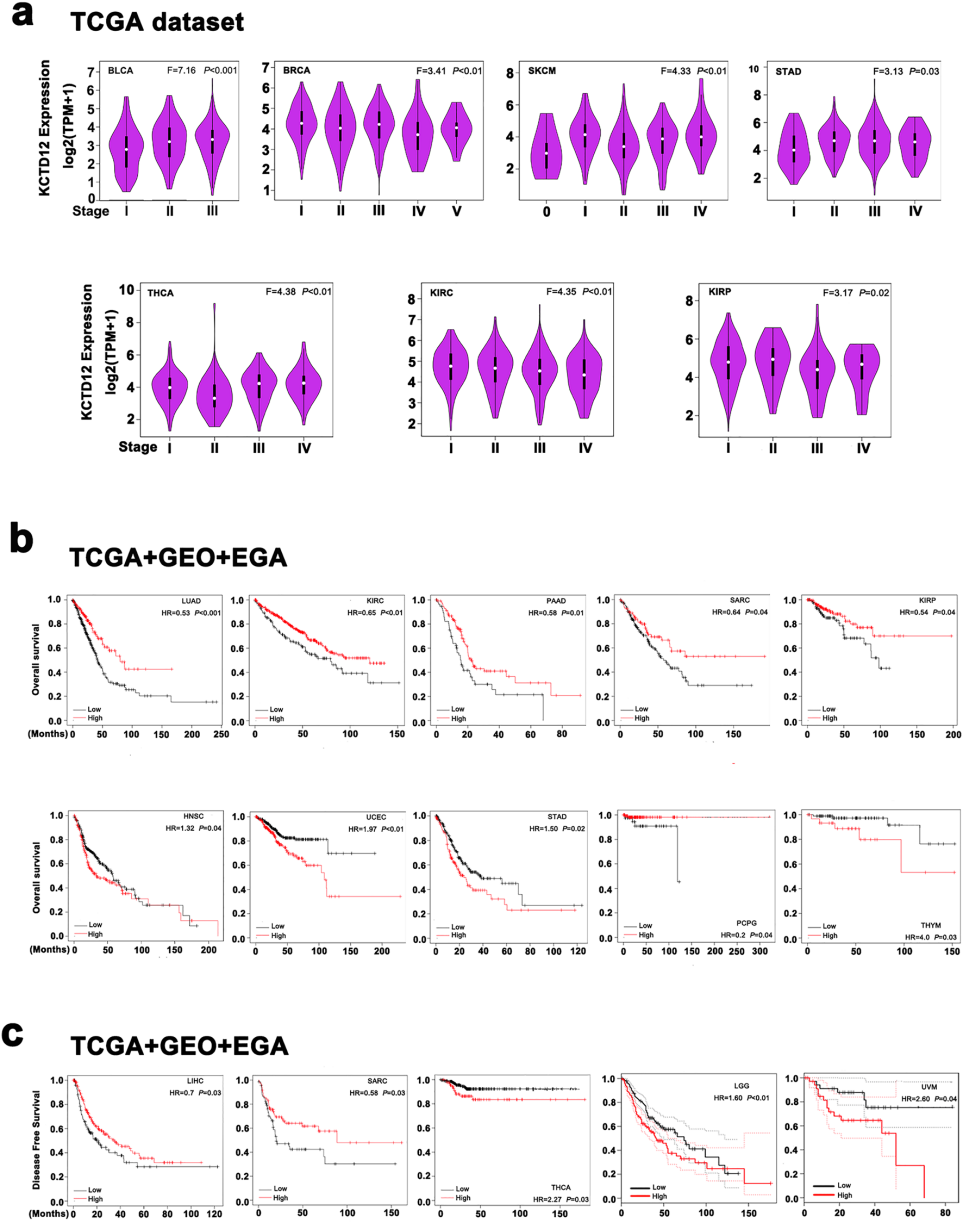
Effect of KCTD12 on different tumor stages and prognoses. (**a**) Expression levels of KCTD12 in tumors of different stages. (**b**,**c**) The relationship between KCTD12 expression and OS, DFS in tumor patients.

We used GEPIA 2 to explore the relationship between KCTD12 expression and tumor stage. KCTD12 levels increased with tumor progression in BLCA, BRCA, SKCM, STAD, and thyroid carcinoma (THCA) (Fig. 2a). In KIRC and KIRP, the expression of KCTD12 decreased with increasing tumor stage (*P* < 0.05).

We analyzed the correlation between KCTD12 and the prognosis of patients with tumors through Kaplan–Meier plotter analysis using TCGA, GEO, and EGA databases. Overall, high KCTD12 expression in LUAD (*P* = 0.000), KIRC (*P* = 0.008), PAAD (*P* = 0.01), sarcoma (SARC, *P* = 0.044), KIRP (*P* = 0.038), and pheochromocytoma and paraganglioma (PCPG, *P* = 0.044) patients was associated with good prognosis, but high KCTD12 expression in HNSC (*P* = 0.043), UCEC (*P* = 0.001), STAD (*P* = 0.015) and thymoma (THYM, *P* = 0.026) patients with poor overall survival (OS) (Fig. 2b). In addition, high levels of KCTD12 were positively correlated with disease-free survival (DFS) in LIHC (*P* = 0.032) and SARC (*P* = 0.026) patients but negatively with LGG (*P* = 0.004), THCA (*P* = 0.034), and uveal melanoma (UVM, *P* = 0.041) (Fig. 2c). These data suggested that the expression of KCTD12 and prognosis vary among different tumors.

### KCTD12 gene mutation analysis data

**Figure 3:**
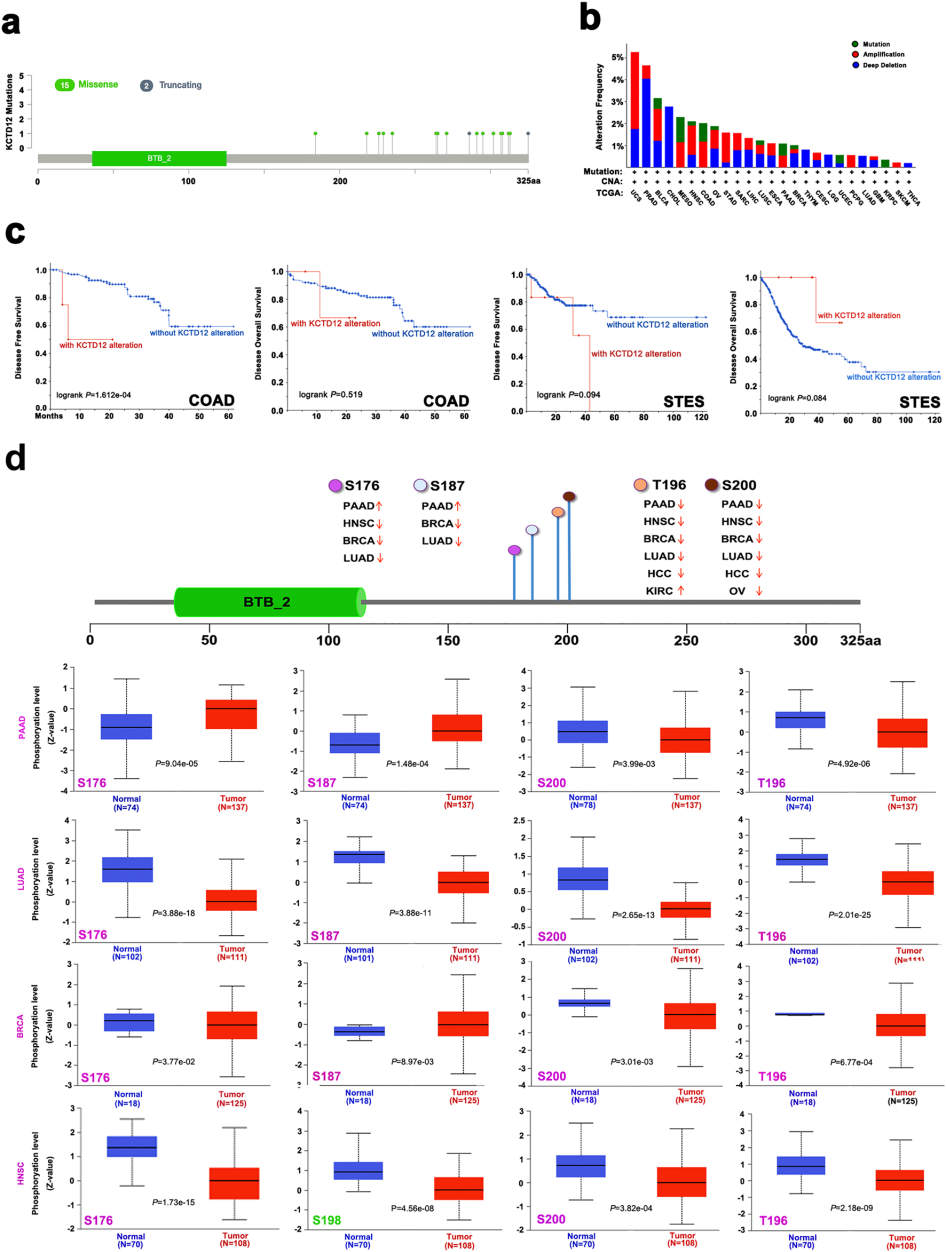
Mutation and phosphorylation analysis in different tumors in the TCGA database. (**a**) KCTD12 mutation site. (**b**) Alteration frequency with mutation type of KCTD12. (**c**) Association of KCTD12 mutation status with OS and DFS in COAD and STES. (**d**) The expression levels of KCTD12 phosphoprotein in PAAD, LUAD, BRCA, and HNSC based on CPTAC (including S176, S187, S200, and T198 sites).

Data from the cBioPortal for cancer genomics showed that the protein encoded by KCTD12 contained 325 amino acids with 15 missense and two truncated mutations (Fig. 3a, Table 1). Mutations in KCTD12 cause changes in the codons encoding amino acids, which may affect the biological function of mutant KCTD12. In uterine carcinosarcoma, CNA mutations are the major mutation type, accounting for more than 3% of the cases. Interestingly, there was a deep deletion in most tumor mutation types. Prostate adenocarcinoma (PRAD) showed deep deletion mutations in 4% of the cases (Fig. 3b). The E325Sfs*46 alteration, which can induce frame-shift mutations in KCTD12, was detected in 3789 BRCA cases that were devoid of base C, and the encoded amino acid was converted from glutamic acid to serine. We further explored the prognostic relationship between KCTD12 mutations and different clinical tumor cases. Mutations in KCTD12 in patients with COAD were associated with poor DFS (*P* = 1.612e−04). Although there was no significant effect on DFS (*P* = 0.094) or OS (*P* = 0.084) (Fig. 3c), KCTD12 had a positive effect.

**Table 1:** The missense and truncated mutation of KCTD12 gene.

Cancer type detailed	Protein change	Mutation type	Copy#	Allele Fre (T)	#Mutation in sample
Serous ovarian cancer	Q308H	Missense	ShallowDel	0.5	43
Lung squamous cell carcinoma	D307Y	Missense	ShallowDel	0.4	306
Bladder urothelial carcinoma	R184H	Missense	Diploid	0.36	320
Bladder urothelial carcinoma	M291I	Missense	ShallowDel	0.11	406
Uterine endometrioid carcinoma	K229R	Missense	Diploid	0.26	10,042
Uterine mixed endometrial carcinoma	K312N	Missense	Diploid	0.08	1560
Breast invasive carcinoma (NOS)	I313T	Missense	Diploid	0.15	24
Breast invasive carcinoma (NOS)	E325Sfs*46	FS del	Gain	0.1	3879
Pancreatic adenocarcinoma	S295N	Missense	Diploid	0.21	14,304
Mucinous adenocarcinoma of the colon and rectum	A226V	Missense	Diploid	0.12	2513
Mucinous adenocarcinoma of the colon and rectum	R235P	Missense	Diploid	0.2	4191
Colon adenocarcinoma	Y271H	Missense	Diploid	0.39	1198
Colon adenocarcinoma	F302Y	Missense	Gain	0.13	149
Colon adenocarcinoma	Y218H	Missense	Gain	0.21	1108
Papillary renal cell carcinoma	R265G	Missense	Diploid	0.3	113
Head and neck squamous cell carcinoma	E264K	Missense	Diploid	0.35	66
Pleural mesothelioma, epithelioid type	E286*	Nonsense	Gain	0.51	53
Uterine endometrioid carcinoma	K229R	Missense	Diploid	0.26	10,042

### Phosphorylation analysis of KCTD12 protein

The CPTAC database was used to compare the phosphorylation levels of KCTD12 in tumors and normal tissues. We analyzed four tumor types: PAAD, LUAD, BRCA, and HNSC. The phosphorylation level of S176 decreased in LUAD (*P* = 3.88e−18), BRCA (*P* = 3.77e−02), and HNSC (*P* = 1.73e−15) tumor tissues, but increased in PAAD (*P* = 9.04e−05) tumor tissues. Similarly, the phosphorylation level of S187 increased in PAAD (*P* = 1.48e−04), decreased in LUAD (*P* = 3.88e−11), and BRCA (*P* = 8.97e−03), and the phosphorylation level of S198 decreased in HNSC (*P* = 4.56e−08). In the four types of tumors explored, the phosphorylation level of KCTD12 at S200 in tumor tissues was significantly lower than that in normal tissues [PAAD (*P* = 3.99e−03), LUAD (*P* = 2.65e−13), BRCA (*P* = 3.01e−03), and HNSC (*P* = 3.82e−04)]. The phosphorylation levels at T196 decreased in all tumors [PAAD (*P* = 4.92e−06), LUAD (*P* = 2.01e−25), BRCA (*P* = 6.77e−04), and HNSC (*P* = 2.18e−09)] (Fig. 3d).

### Immune infiltrate analysis data

**Figure 4:**
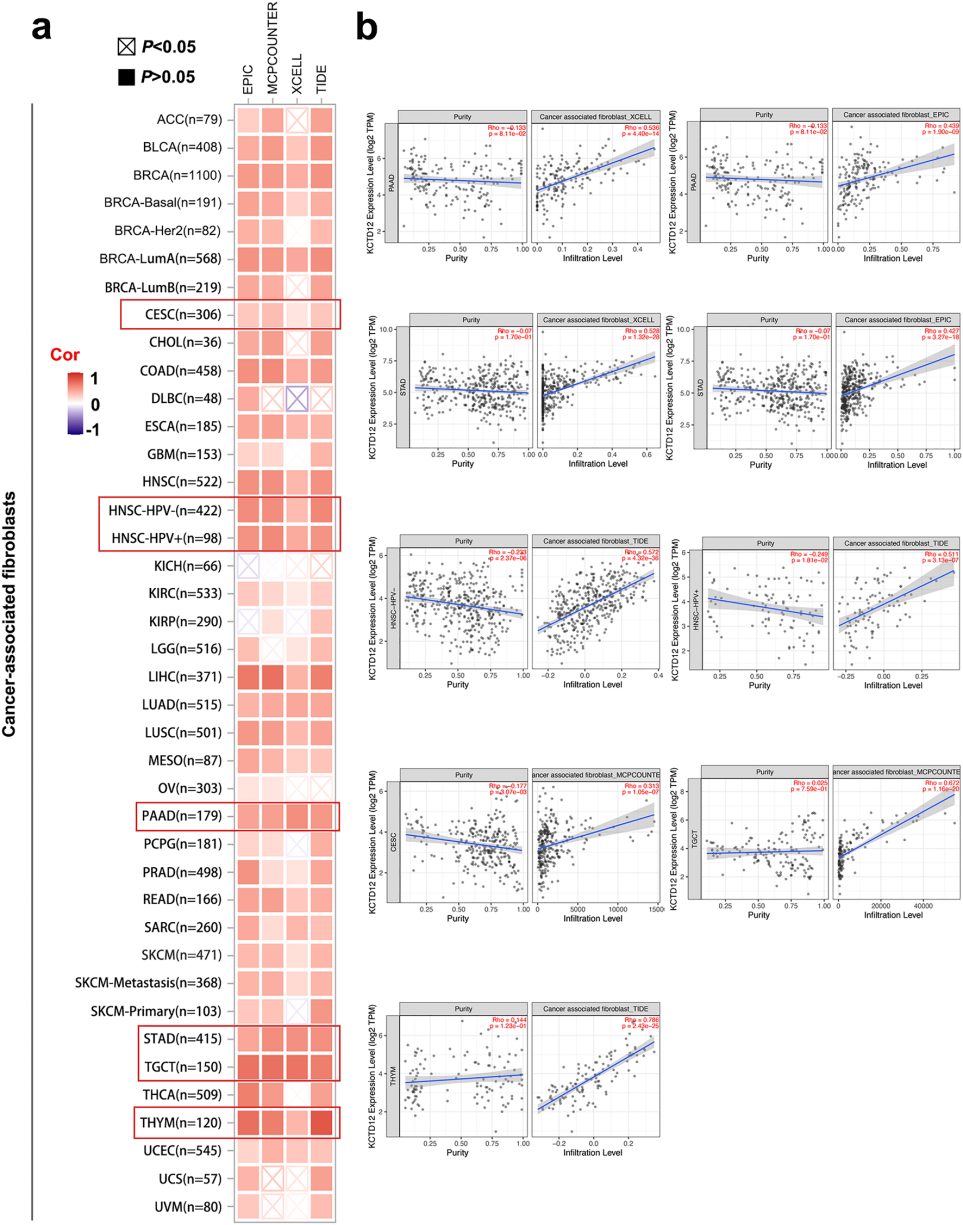
Infiltration of CAFs in different tumors. (**a**) Different algorithms based on the cBioPortal showed the infiltration level of CAFs in various tumors. (**b**) The infiltration level of CAFs in CESC, PAAD, STAD, TGCT, and THYM was positively correlated with KCTD12 expression.

**Figure 5:**
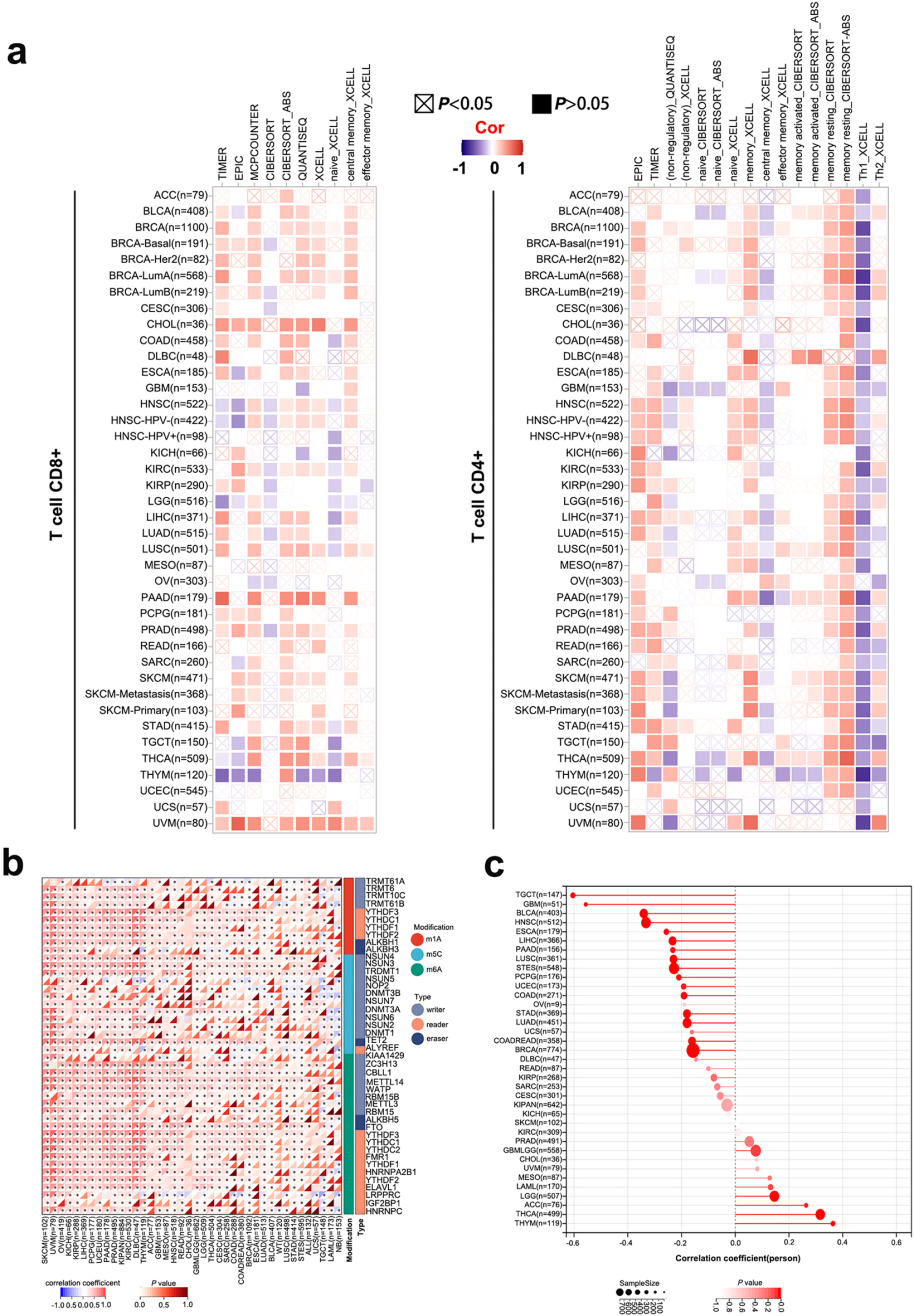
Analysis of KCTD12 and immune cell infiltration. (**a**) Correlation of the infiltration degree of CD8^+^ T and CD4^+^ T cells with KCTD12 according to the cBioPortal. The CD8^+^ T cell infiltration levels in THYM were negatively correlated with KCTD12 (left panel), and KCTD12 expression was negatively correlated with CD4^+^ T cells in all tumors (right panel). (**b**) Correlation analysis of RNA gene modifications (m6A, m5C, and m1A) with KCTD12. (**c**) Correlation analysis between KCTD12 expression and tumor stemness.

Tumor immunosuppression, metastasis, drug resistance, and other aspects of the tumor microenvironment (TME) have recently received high attention. The TME is mainly composed of tumor cells and infiltrating immune cells such as cancer-associated fibroblasts (CAFs)^9^. In addition, dynamic interactions between cancer cells, TME cells, and non-cellular components are crucial for generating heterogeneity, clonal evolution, and enhancing multiple drug resistance in tumor cells^10^. CAFs are also involved in regulating tumor immune cell infiltration^11^. As shown in Fig. 4, the expression of KCTD12 in CESC, PAAD, STAD, testicular germ cell tumors (TGCT), THYM, and HNSC (HNSC-HPV−/HNSC-HPV+) TCGA tumors showed a statistically positive correlation with the degree of CAF infiltration. We further used the TIMER 2.0 portal combined with the EPIC, TIMER, QUANTISEQ CIBERSORT, CIBERSORT-ABS, XCELL, MCPCOUNTER, and TIDE algorithms to investigate the immune cell infiltration level and its potential relationship with KCTD12 gene expression. There was a negative correlation between the CD8^+^ T cell infiltration levels of immune cells and KCTD12 expression in most algorithms for THYM, LGG, and HNSC (Fig. 5a, left panel). Interestingly, when we analyzed CD^+^ 4 T cells, we found that the infiltration level of CD^+^ 4 T cells (Th1 and central memory cells) significant decreased in almost all tumors using the XCELL algorithm (Fig. 5a, right panel).

### RNA modification and tumor stemness analysis data

From the TCGA data, m6A RNA modification was the most common modification in all tumors, and most of the m6A-methylated marker genes were positively correlated with the expression of KCTD12 in tumors. For example, in PRAD, all m6A-methylated marker genes positively correlated with the expression of KCTD12 (Fig. 5b).

We identified 37 cancer species and calculated the Pearson correlations for each tumor. Significant correlations were observed in 20 tumors, including positive correlations in 4 tumors [LGG, THYM, THCA, and adrenocortical carcinoma (ACC)] and negative correlations in 16 tumors [GBM, LUAD, COAD, colorectal adenocarcinoma (COADREAD), BRCA, esophageal carcinoma (ESCA), STES, STAD, UCEC, HNSC, LUSC, LIHC, PAAD, TGCT, PCPG, and BLCA] (Fig. 5c, Table 2). These results indicate that up-regulated expression of KCTD12 is negatively correlated with tumor stemness in most types of tumors.

**Table 2:** KCTD12 affects the stemness in different tumors.

Cancer type	n	R	P value
LGG	507	0.145569367691029	0.00101199161613922
THYM	119	0.363844642143469	4.75203E−05
THCA	499	0.31641223726991	4.58E−13
ACC	76	0.263749763054007	0.0213275035926418
GBM	51	− 0.556574051390226	2.22369E−05
LUAD	451	− 0.179329510599078	0.000128721467289359
COAD	271	− 0.190837059481391	0.00159889255676361
COADREAD	358	− 0.160897805716247	0.00226112166325264
BRCA	774	− 0.157131203951344	1.12432E−05
ESCA	179	− 0.256665847335439	0.000523993346980214
STES	548	− 0.22812009354371	6.69E−08
STAD	369	− 0.179756176916682	0.000521251852797944
UCEC	173	− 0.192158528617272	0.0113162944825093
HNSC	512	− 0.332346115922799	1.14E−14
LUSC	361	− 0.22985170361425	1.02781E−05
LIHC	366	− 0.233520807756285	6.33743E−06
PAAD	156	− 0.232563408202342	0.00348326235057261
TGCT	147	− 0.604484094915386	5.19E−16
PCPG	176	− 0.209842574780856	0.0051862723892506
BLCA	403	− 0.340752282074657	2.05E−12

### Genomic heterogeneity and KCTD12 expression analysis data

**Figure 6:**
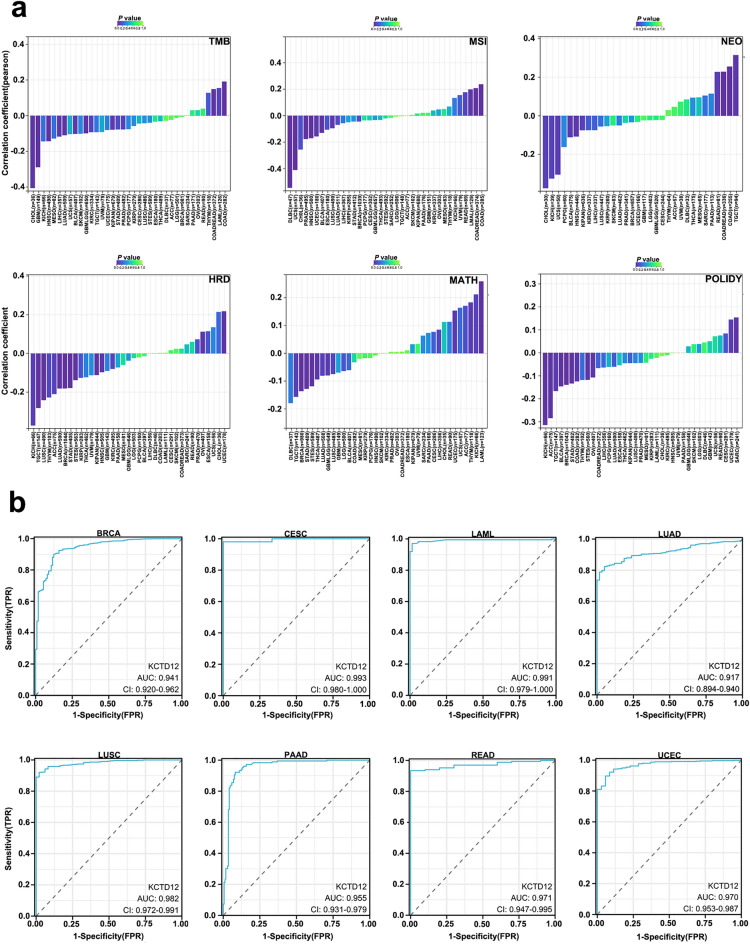
KCTD12 genomic heterogeneity and ROC diagnostic curve. (**a**) Correlation of KCTD12 with TMB, MSI, NEO, HRD, MATH, and PLOIDYS in multiple malignancies. (**b**) ROC curve to assess the sensitivity of KCTD12 for tumor diagnosis.

Subclonal components, consisting of genes driven by varying degrees of genetic alterations, are present in the vast majority of solid tumors, and genomic heterogeneity responses in tumors manifest as phenotypic diversity at the cellular level. TMB is considered a predictive biomarker of the response to immune checkpoint blockade, based on the fact that certain mutations increase the expression of multiple antigenic peptides, thereby enhancing immunogenicity^12^. It is used to assess the sensitivity of tumor immunotherapy. The expression of KCTD12 with genomic heterogeneity showed that the expression of KCTD12 in COAD (*R* = 0.191, *P* = 0.001) and COADREAD (*R* = 0.149, *P* = 0.004) positively correlated with TMB. A negative correlation was observed between KCTD12 and TMB in GBM (*R* = − 0.289, *P* = 0.000), LUAD (*R* = − 0.110, *P* = 0.0131), HNSC (*R* = − 0.143, *P* = 0.001), CHOL (*R* = − 0.410, *P* = 0.016), and other tumors (Fig. 6a). Other indicators for the assessment of KCTD12 genomic heterogeneity tumor mutation burden (TMB), microsatellite instability (MSI) score, neoantigen (NEO), homologous recombination deficiency (HRD), mutant-allele tumor heterogeneity (MATH) and PLOIDYS data, are presented in Table 3 and Fig. 6a.

**Table 3:** Relationship between KCTD12 gene expression and genomic heterogeneity.

Cancer type	TMB	MSI	NEO	HRD	MATH	POLIDY
COAD	R = 0.191, P = 0.001	R = 0.238, P = 0.000	R = 0.256, P = 0.000	–	–	R = − 0.124, P = 0.037
COADREAD	R = 0.149, P = 0.004	R = 0.208, P = 0.000	R = 0.229, P = 0.000	–	–	–
GBM	R = − 0.289, P = 0.000	–	–	–	–	–
GBMLGG	R = − 0.010, P = 0.012	–	–	–	R = − 0.080, P = 0.043	–
LUAD	R = − 0.110, P = 0.013	–	–	R = − 0.181, P = 0.000	–	–
KIPAN	R = − 0.079, P = 0.040	–	–	R = − 0, 112, P = 0.006	–	–
HNSC	R = − 0.143, P = 0.001	R = − 0.170, P = 0.000	R = − 0.108, P = 0.034	R = − 0.100, P = 0.031	–	–
LIHC	R = − 0.117, P = 0.027	–	–	–	–	–
BLCA	R = − 0.102, P = 0.039	R = − 0.130, P = 0.008	R = − 0.112, P = 0.040	–	–	R = − 0.146, P = 0.004
CHOL	R = − 0.410, P = 0.016	–	–	–	–	–
LAML	–	R = 0.199, P = 0.024	–	–	R = 0.259, P = 0.004	–
PRAD	–	R = − 0.177, P = 0.000	–	–	–	–
UCEC	–	R = − 0.157, P = 0.035	–	R = 0.218, P = 0.009	R = 0.154, P = 0.042	–
LUSC	–	R = − 0.095, P = 0.035	–	R = − 0.242, P = 8.16e−8	–	–
UCS	–	R = − 0.412, P = 0.001	–	–	–	–
DLBC	–	R = − 0.547, P = 0.000	–	–	–	–
TGCT	–	–	R = 0.315, P = 0.026	R = − 0.282, P = 0.000	–	R = − 0.166, P = 0.044
OV	–	–	–	R = 0.112, P = 0.023	–	R = − 0.107, P = 0.030
BRCA	–	–	–	R = − 0.181, P = 5.96e−9	R = − 0.136, P = 0.000	R = − 0.139, P = 0.000
STES	–	–	–	R = − 0.138, P = 0.002	R = − 0.119, P = 0.004	R = − 0.117, P = 0.005
STAD	–	–	–	R = − 0.179, P = 0.000	R = − 0.128, P = 0.010	R = − 0.133, P = 0.007
THYM	–	–	–	–	R = 0.184.P = 0.047	–
THCA	–	–	–	–	R = − 0.094, P = 0.038	–
SARC	–	–	–	–	–	R = 0.154, P = 0.017
ACC	–	–	–	–	–	R = − 0.284, P = 0.013
KICH	–	–	–	–	–	R = − 0.314, P = 0.010

### ROC curve analysis of KCTD12 for tumor diagnosis

Receiver operating characteristic (ROC) curves were used to assess the specificity and sensitivity of KCTD12 in the diagnosis of multiple tumors. The area under the curve (AUC) was used as a diagnostic indicator, and the AUCs of the ROC curve of KCTD12 in various tumors were as follows: BRCA (*AUC*: 0.941, *CI*: 0.920–0.962), CESC (*AUC*: 0.993, *CI*: 0.980–1.000), LAML (*AUC*: 0.991, *CI*: 0.979–1.000), LUAD (*AUC*: 0.917, *CI*: 0.894–0.940), (*AUC*: 0.982, *CI*: 0.972–0.991), LUAD (*AUC*: 0.955, *CI*: 0.931–0.979), READ (*AUC*: 0.971, *CI*: 0.947–0.995), (*AUC*: 0.970, *CI*: 0.953–0.987) (Fig. 6b).

### Functional analysis of KCTD12 and its related genes

**Figure 7:**
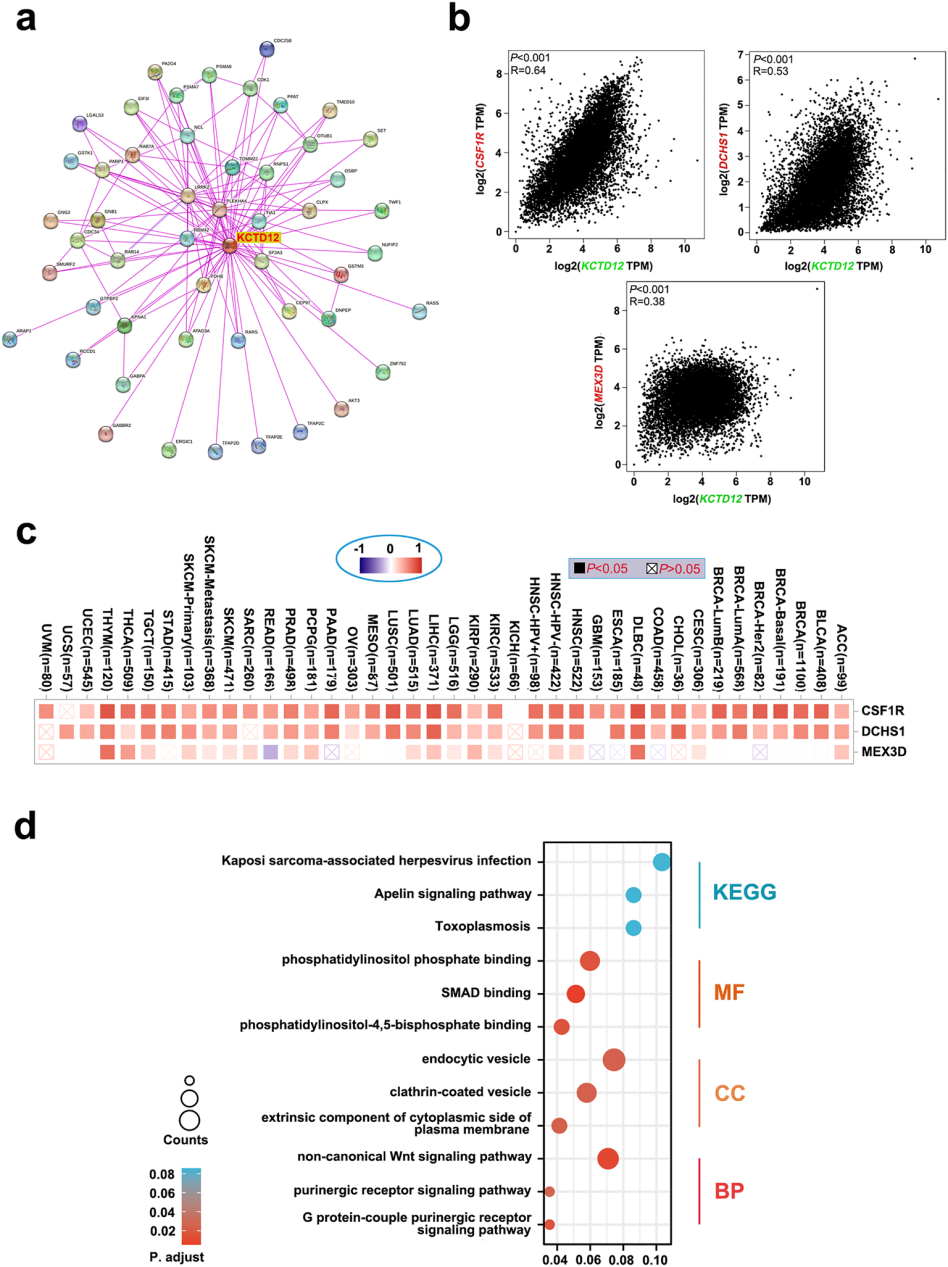
Functional analysis of the KCTD12 gene. (**a**) Top 50 proteins that interact with KCTD12 as identified by STRING analysis. (**b**) Among the 100 genes related to KCTD12 in GEPIA 2, the correlation degree of three genes (CSF1R, DCHS1, and MEX3D) with KCTD12 was analyzed. (**c**) The corresponding heatmap in (**b**) was generated using TIMER 2.0. (**d**) KEGG/GO was used to analyze the function of the KCTD12 gene in cells and its possible involvement in signaling pathways [Kanehisa Laboratories (2022). KEGG Database. Retrieved August 9, 2023, from http://www.kegg.jp/kegg/kegg1.html].

To further explore the molecular mechanism of KCTD12 in tumors, we identified 50 encoded proteins that interacted with KCTD12 based on experimental verification using the STRING portal. In addition, we mined 100 genes that correlated with KCTD1 using the GEPIA 2 data portal and generated a protein interaction network diagram using the proteins that interacted with KCTD12 (Fig. 7a). Among the top 100 genes related to KCTD12, we analyzed the following three genes in different tumors, all of which were positively correlated with KCTD12: colony-stimulating factor 1 receptor (CSF1R, *R* = 0.64, *P* < 0.001), dachsous cadherin-related 1 (DCHS1, *R* = 0.53, *P* < 0.001), and Mex-3 RNA-binding family member D (MEX3D, *R* = 0.38 and *P* < 0.001) (Fig. 7b). The heat map also showed that KCTD12 was positively associated with these three genes in most cancer types, including ACC, DLBC, KIRP, LIHC, and THYM (Fig. 7c).

Finally, we used the obtained 150 genes for enrichment analysis. KEGG analysis showed that KCTD12-related genes were mainly enriched in Kaposi sarcoma-associated herpes virus infection, the apelin signaling pathway, and toxoplasmosis. GO enrichment analysis showed that most of these genes were related to purine metabolism pathways, purinergic receptors, and G protein-coupled purinergic receptors (Fig. 7d, Table S1).

## Discussion

As one of the components of the GABA_B_R, KCTD12 mainly interacts with the activated receptor to induce K^+^ desensitization^13^. Phosphorylation of S892 on protein kinase A induces conformational changes in the GABA_B_R/KCTD12 complex, thereby slowing the desensitization reaction induced by KCTD12. In contrast, the binding of KCTD12 promotes the phosphorylation of S892^3^. Recent studies on electrical and chemical synapses in cancer have shown that cancer is an electrically active entity, the ion channel acts as a central regulator of cell electrical properties and is involved in all steps of tumorigenesis^14^. Recently, several studies have investigated the role of KCTD12 in cancer. Increased KCTD12 expression regulates the cell cycle and promotes tumor occurrence, however, up-regulation of KCTD12 inhibits the growth of COAD, UVM, and BRCA cells^13, 15^. Recent oncology studies have shown that KCTD12 plays an important role in maintaining stemness and promoting or inhibiting tumor cell proliferation^4^. Therefore, it is unclear whether the effect of KCTD12 on tumors is consistent. To date, there have been no comprehensive studies on KCTD12 across cancers. In this study, we comprehensively analyzed the role of KCTD12 expression in the prognosis, phosphorylation, RNA modification, immune infiltration, and genomic heterogeneity of different tumors using TCGA, GTEx, CPTAC, and other databases.

The ultimate goal of investigating the abnormal expression of genes in tumors is to explore the influence of genes on the survival of patients with tumors. KCTD12 has been widely studied in gastrointestinal stromal tumors (GIST). The expression of KCTD12 is specific to GIST and can be used as a predictor of GIST recurrence. Moreover, KCTD12 deletion significantly correlated with GIST recurrence, and KCTD12 contributed to the prevention of GIST metastasis^16–18^. KCTD12 is an emerging biomarker for sarcoma that has been demonstrated by immunohistochemistry in multi-center studies on patients with clinical sarcoma^19^. KCTD12 can combine with miRNAs as the information transmission axis component of LINC00365 to regulate glycolysis in LUAD cells, thereby affecting the progression of LUAD^20^. KCTD12 inhibits the proliferation and invasion of UVM and ESCA as well as the growth of xenograft tumors. Low level of KCTD12 enhances tumor stemness in melanoma^4, 6^. Low expression of KCTD12 in COAD is associated with poor prognosis and is an independent risk factor for short DFS and OS in patients with COADREAD because it promotes the ERK pathway^6^. In BRCA, down-regulation of KCTD12 expression can enhance G1/S transition by activating AKT/FOXO1 signaling, thereby significantly promoting cell proliferation and tumorigenesis in vitro and predicting poor prognosis^7^. In marked contrast, KCTD12 expression is elevated in CESC and LUAD. As a carcinogenic factor, KCTD12 binds to CDC25B and activates CDK1 and Aurora A, which promotes the G2/M transition and facilitates tumorigenesis. Aurora A phosphorylates serine 243 of KCTD12, triggering a positive feedback loop that enhances the effects of KCTD12. In terms of prognosis, LUAD patients with high expression of KCTD12 have a shorter survival^15^. In the present study, the expression of KCTD12 was down-regulated in most tumors, however, the same expression trend of KCTD12 was observed with different outcomes in cancer. The low expression of KCTD12 protein in LUAD and HNSC was negatively associated with the OS of patients with LUAD but positively associated with the OS of patients with HNSC. High KCTD12 protein expression was not associated with prognosis of PAAD patients. In LIHC, KCTD12 exhibited a protective effect on DFS. We used the SangerBox 3.0 data portal to verify the influence of KCTD12 expression on the prognosis of patients by Cox regression analysis, and found no significant difference in the influence of KCTD12 expression on the prognosis of the above three tumors, which may because the clinical data were from different databases. Therefore, further studies are required to confirm the effect of KCTD12 on the prognosis of patients with tumors.

Phosphorylation of approximately 30% of human proteins is reported to be involved in almost all cellular and physiological processes, such as cell division, protein breakdown, signal transduction, gene expression regulation, and protein interactions^21^. Mutations in protein phosphorylation may lead to the occurrence and development of malignant tumors, whereas abnormal protein phosphorylation may lead to abnormal cellular activities and eventually cancer^22^. In the present study, the phosphorylation levels of S200 and T196 reduced in various tumors, and the mutation frequency of KCTD12 in COAD was up to 2%, which significantly shortened DFS in patients with COAD. In addition, the expression of KCTD12 is correlated with RNA modification and negatively correlated with the stemness of various tumors, which can reduce the malignant phenotype of tumor cells.

In LUAD, KCTD12 expression positively correlated with CD4^+^ T cells, CD8^+^ T cells, neutrophils, and macrophages^23^, which is consistent with our findings. In our study, the expression of KCTD12 was closely correlated with the infiltration of CD8^+^ and CD4^+^ cells in most tumors, except for THYM, and the infiltration level of CAFs was positively correlated with KCTD12 expression in all tumors, which opens up the possibility of immunotherapy for tumors. Interestingly, our results on KCTD12 expression showed that the trend of mRNA levels and protein levels were significantly opposite in PAAD, and the status of high KCTD12 expression was favorable to patient prognosis. Unlike other types of tumors, the phosphorylation level of KCTD12 at S176 and S187 was significantly elevated, the level of CAFs and CD8^+^ T cell infiltration in PAAD was significantly increased, and the diagnostic specificity for PAAD was high. The unique TME of PAAD can generate strong resistance to chemotherapy while also helping tumor cell immune escape and immune therapy fatigue. Therefore, the development of targeted drug therapies for PAAD that target CAFs is currently a research hotspot, and some drugs have entered the clinical trial stage^24^.

Tumor cell stemness analysis indicated that KCTD12 was negatively correlated with stemness in most malignant tumors, providing evidence that KCTD12 is an oncogene. Tumor heterogeneity can affect TBM, which further affects the response to immune checkpoint inhibitor treatment^25^. Genomic heterogeneity analysis allowed us to evaluate the expression of KCTD12 in GBM, COAD, CHOL, and other tumors. Our study shows that patients with KCTD12 mutations had shorter DFS and high TMB in COAD, while the high abundance of CD8^+^ T cells suggests that immunotherapies such as PD-1/PD-L1 monoclonal antibodies may be of greater potential value for patients with KCTD12 mutated COAD. Therefore, elevation or decrease of KCTD12 can be used as a sensitive biomarker for the diagnosis of malignant tumors.

In summary, we explored the relationship between KCTD12 expression and tumor clinical stage, prognosis, mutation, genomic heterogeneity, phosphorylation, and immune infiltration using pan-cancer analysis from a new perspective, providing information to better understand the significance of KCTD12 in multiple tumors. The present study revealed a correlation between the KCTD12 gene and pan-cancer, indicating an important role for KCTD12 in various tumors through multiple signaling pathways.

## Materials and methods

### Gene expression analysis

First, the mRNA expression levels of KCTD12 in 33 tumors, normal tissues, and specific tumor subtypes were retrieved using Tumor Immune Estimation Resource version 2.0 (TIMER2.0, website: http://timer.cistrome.org/)^26^, and the KCTD12 gene name was entered from Gene DE TAB. Through the gene expression analysis function option of Gene Expression Profiling Interactive Analysis 2 (GEPIA 2^27^, “Expression DIY” was selected, and “Box Plot” was then selected. TCGA normal and GTEx data were matched, and the mRNA expression of KCTD12 in some cancer species was plotted to supplement the data in TIMER2.0.

We then used The University of Alabama at Birmingham Cancer data analysis portal (UALCAN portal; website: http://ualcan.path.uab.edu/) to explore the protein expression of KCTD12 in various tumors using the Clinical Proteomic Tumor Analysis Consortium (CPTAC)^28^. We determined the protein expression levels of KCTD12 in BRCA, OV, COAD, LUAD, LIHC, HNSC, PAAD, and GBM. We also determined the phosphorylation levels of KCTD12, including phosphorylation at S176, S185, S187, S198, T196, and S200 in different tumors from the CPTAC database.

### Pathological stage and survival prognosis analysis

Similarly, we used the gene expression function of the GEPIA 2 portal to input the name of the KCTD12 gene in the “stage plot” interface, and we used the major stage for plotting. We selected and entered the corresponding tumor types and then obtained the pathological stage plot. Kaplan–Meier plotter data from GEO, EGA, and TCGA were used to analyze the expression of KCTD12 and the prognosis of different tumors^29^. The KCTD12 gene name was input into the mRNA-seq pan-cancer interface, and auto-select was used as the cutoff to select all tumor types to draw the overall survival and disease-free survival Kaplan–Meier plots.

### Gene mutation analysis

The cBio For Cancer Genomics Portal (cBioportal, website: https://www.cbioportal.org/) was used to analyze the correlation between KCTD12 mutations and the survival of patients with different tumors^30^. We input the name of the KCTD12 gene into the “Quick Search Beta!” interface, and the distribution of KCTD12 in different tumors was obtained. These items included gene mutation frequency, mutation type, and gene copy number. We next selected the tumor type in the left column of the interface in “Query,” and we selected “TCGA, Pan-cancer Atlas” in the middle column. We selected “Mutations” in the “Query By Gene” interface and entered KCTD12 in “Enter Genes” and selected “Comparison/Survival”. We then selected “Survival” to obtain the influence of KCTD12 mutation on the prognosis of patients, including overall survival and disease-free survival.

### Immune infiltration analysis

In TIMER 2.0, we selected “Immune” and entered KCTD12 in the “Gene Expression” column, and “T cell CD8^+^”, “T cell CD4^+^” and “Cancer-associated fibroblast” were selected in the “Immune Infiltrates”-associated fibroblast column. We selected CD4^+^/CD8^+^ T cell (EPIC, TIMER, QUANTISEQ CIBERSORT, CIBERSORT-ABS, and XCELL) and cancer-associated fibroblast (EPIC, MCPCOUNTER XCELL, and TIDE) indices to evaluate immune infiltration, such as color, to express the degree of infiltration depth (red or blue). The results were presented as heat maps.

### Analysis of RNA-modified genes

The m6A and m5C m1A RNA modifications contribute to cancer occurrence and progression of cancer^31^. We downloaded the following standardized pan-cancer dataset from the UCSC database (https://xenabrowser.net/): TCGA TARGET GTEx (PANCAN, N = 19,131, G = 60,499). Furthermore, we extracted the expression data of ENSG00000178695 (KCTD12) and 44 marker genes of Class III RNA modifications [m1A(10), m5C(13), and m6A(21)] in each sample. We screened the following sample sources: primary solid tumors, primary tumors, primary blood-derived cancer bone marrow, and primary blood-derived cancer peripheral blood samples. We also filtered all the normal samples and performed a log2(x + 0.001) transform for each expression value. We then calculated Pearson’s correlations between ENSG00000178695 (KCTD12) and the five immune pathway marker genes.

### Analysis of tumor stemness and expression of KCTD12

Currently, it is generally accepted that all tumors have cancer stem cells (CSCs) and that cancer stemness can be acquired through epithelial-mesenchymal transformation (EMT) procedures or by eliminating senescence, resulting in drug resistance, angiogenesis, invasion, and metastatic potential of tumor cells^32^. We downloaded the following standardized pan-cancer dataset from the University of California Santa Cruz (UCSC) database: TCGA Pan-Cancer (PANCAN, N = 10,535, G = 60,499). We further extracted ENSG00000178695 (KCTD12) gene expression data from each sample and screened the sample sources as primary blood-derived cancer-peripheral blood and primary tumor samples, which we obtained from a previous study^33^. For each tumor, we calculated the DNA methylation characteristics (DNAss) by integrating the dryness index and gene expression data of the samples, and further performed log2(x + 0.001) transformation for each expression value. Finally, cancer species with fewer than three samples from a single cancer species were eliminated.

### KCTD12 genomic heterogeneity and gene expression analysis

Similar to 2.5, we downloaded the uniformly normalized pan-cancer dataset from the UCSC database through SangerBox Data Portal Analysis (http://sangerbox.com), from which we extracted the expression data of ENSG00000178695 (KCTD12) in each sample. In addition, TMB, MSI score, NEO, HRD, MATH, and PLOIDYS data were calculated for each tumor using the infer heterogeneity function, and the expression data of KCTD12 in 37 cancer species were obtained.

### Diagnostic significance of KCTD12 in tumors

To evaluate the diagnostic efficacy of KCTD12 for various tumor types, we plotted ROC curves and calculated the AUC using patient clinical data from TCGA through the Xiantao Academic Data Platform (https://www.xiantao.love).

### Functional analysis of KCTD12 and related genes

STRING version 11.5 is a database of protein–protein interaction networks (https://cn.string-db.org/)^34^. We selected “Protein by name” in the left column of the STRING database search interface and input KCTD12, and we selected Homo sapiens as the species. We also selected “full STRING network” as the network type, and we selected evidence in the network edges. We also selected experiments on active interaction sources and set the minimum required interaction score to a low confidence (0.150) to allow for the maximum number of interactors to be displayed. We selected “no more than 50 interactors,” and after updating the parameter settings, 50 proteins of KCTD12 interaction were obtained.

We input KCTD12 into “Similar Genes Detection” in GEPIA 2 and selected the top 100 similar genes. After selecting “Used Expression Datasets” in the tumor category from “TCGA Tumor,” we obtained the first 100 genes associated with KCTD12. The first 20 genes among the top 100 KCTD12-related genes obtained from GEPIA 2 were selected, and the “Correlation Analysis” function of GEPIA 2 was used to analyze their correlation with KCTD12. We further explored the correlation between the first several genes and immune cell infiltration using the TIMER 2.0 database. The KEGG and GO database were used to analyze 150 genes (from STRING and TIMER 2.0) to explore the molecular functions of KCTD12 and the potentially involved signaling pathways.

### Statistical analyses

Differences in mRNA expression of KCTD12 between various types of tumors and adjacent normal tissues were analyzed using the Wilcoxon test. The Z-test was used to compare KCTD12 protein expression and phosphorylation levels between the tumor and normal groups in various cancer types. In the correlation analysis between KCTD12 and tumor staging, ANOVA was used for statistical analysis, and the criteria were set as cutoff values: |log2FC| = 1 and q-value = 0.01, using log2(TPM + 1) for the log scale. The EPIC, MCPCOUNTER XCELL, and TIDE algorithms were used to estimate immune infiltration in the correlation analysis between KCTD12 and immune infiltration levels, and the p-values and partial correlations were obtained using the Spearman rank correlation test (purity-adjusted). Pearson’s correlation analysis was used to explore the correlation between KCTD12 and RNA-modified genes, tumor stemness, and genomic heterogeneity. All statistical differences in this study were defined as *P < 0.05, **P < 0.01, ***P < 0.001.

### Supplementary Information

Supplementary Figure S1.

Supplementary Information.

Supplementary Table S1.

## Data Availability

The datasets generated and/or analysed during the current study are available in the TIMER2.0 [TIMER (shinyapps.io)], GEPIA [GEPIA (Gene Expression Profiling Interactive Analysis) (cancer-pku.cn)], UALCAN [UALCAN (uab.edu)], Kaplan–Meier Plotter [Kaplan–Meier plotter (kmplot.com)], cBioPortal [cBioPortal for Cancer Genomics], SangerBox 3.0 [SangerBox], String [STRING: functional protein association networks (string-db.org)], KEGG [KEGG: Kyoto Encyclopedia of Genes and Genomes], Xiantao Academic [xiantaozi.com] repository. The datasets used and/or analysed during the current study available from the corresponding author on reasonable request.
