# Supplementary figures and images for: A pan-cancer analysis of potassium channel tetramerization domain containing 12 in human cancer
Source: Sci Rep. 2023 Aug 25;13:13898. doi: 10.1038/s41598-023-41091-8 (PMC10457314; doi:10.1038/s41598-023-41091-8)

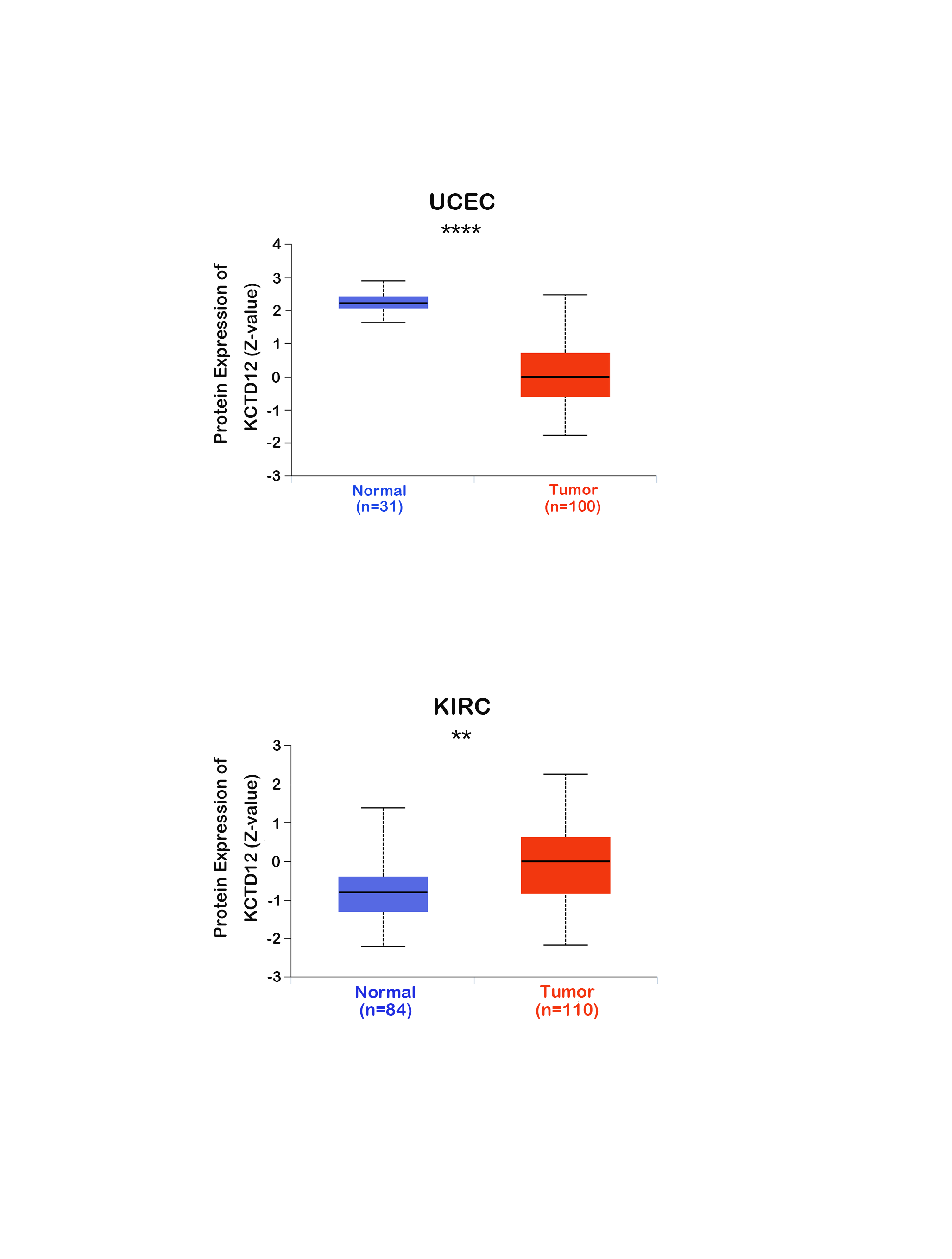

Supplement: Supplementary file 1 — Supplementary Figure S1. [file 41598_2023_41091_MOESM1_ESM.png]
