# Supplementary material for: A pan-cancer analysis of potassium channel tetramerization domain containing 12 in human cancer
Source: Sci Rep. 2023 Aug 25;13:13898. doi: 10.1038/s41598-023-41091-8 (PMC10457314; doi:10.1038/s41598-023-41091-8)
Supplement: Supplementary file 2 — Supplementary Information. [file 41598_2023_41091_MOESM2_ESM.pdf]

The expression levels of KCTD12 protein in UCEC and KIRC in the CTPAC database.
